# Circulating miRNAs in Breast Cancer Diagnosis and Prognosis

**DOI:** 10.3390/cancers14092317

**Published:** 2022-05-07

**Authors:** Barbara Cardinali, Roberta Tasso, Patrizia Piccioli, Maria Chiara Ciferri, Rodolfo Quarto, Lucia Del Mastro

**Affiliations:** 1 Department of Medical Oncology, U.O. Clinica di Oncologia Medica, IRCCS Ospedale Policlinico San Martino, 16132 Genova, Italy; patrizia.piccioli@hsanmartino.it (P.P.); lucia.delmastro@hsanmartino.it (L.D.M.); 2 Department of Experimental Medicine (DIMES), University of Genova, 16132 Genova, Italy; roberta.tasso@unige.it (R.T.); mariachiara.ciferri@outlook.it (M.C.C.); rodolfo.quarto@unige.it (R.Q.); 3 Cellular Oncology Unit, IRCCS Ospedale Policlinico San Martino, 16132 Genova, Italy; 4 Department of Internal Medicine and Medical Specialties (DIMI), University of Genova, 16132 Genova, Italy

**Keywords:** miRNA, breast cancer, biomarkers, liquid biopsy, extracellular vesicles

## Abstract

**Simple Summary:**

In the era of precision medicine, new tools for better management of breast cancer patients are still needed. From this perspective, circulating microRNA (miRNA), a small group of non-coding endogenous RNAs with a nucleotide length of 18–25, could be potentially appealing as diagnostic, prognostic and predictive biomarkers. miRNAs have been found to be deregulated in different pathological conditions including cancers. Moreover, due to their high stability in biological fluid, where they circulate freely or are associated with extracellular vesicles (EVs), they are ideal candidates to be used for non-invasive detection and monitoring. This review highlights the recent findings related to free and EV-derived miRNA applicability in breast cancer, pointing out the advantages, but also the issues, that still limit their translation in clinical practice.

**Abstract:**

Great improvement has been made in the diagnosis and therapy of breast cancer patients. However, the identification of biomarkers for early diagnosis, prognosis, therapy assessment and monitoring, including drug resistance and the early detection of micro-metastases, is still lacking. Recently, circulating microRNAs (miRNAs), circulating freely in the blood stream or entrapped in extracellular vesicles (EVs), have been shown to have a potential diagnostic, prognostic or predictive power. In this review, recent findings are summarized, both at a preclinical and clinical level, related to miRNA applicability in the context of breast cancer. Different aspects, including clinical and technical challenges, are discussed, describing the potentialities of miRNA use in breast cancer. Even though more methodological standardized studies conducted in larger and selected patient cohorts are needed to support the effective clinical utility of miRNA as biomarkers, they could represent novel and accessible tools to be transferred into clinical practice.

## 1. Introduction

Breast cancer (BC) remains the most common malignancy worldwide and the major cause of cancer death among women [1,2]. Currently, BC classification and therapy assessment are mainly based on tumor staging/grading and the following molecular biomarkers: estrogen receptor (ER), progesterone receptor (PgR), human epidermal growth factor receptor 2 (HER2) and Ki-67 (proliferation marker). Based on their expression, BC is classified in four main subtypes that differ in terms of prognosis and outcome: luminal A (ER+, PR+, HER2−, Ki-67 low), luminal B (ER+, PR+/−, HER2+/−, Ki-67 high), HER2 overexpression (ER−, PR−, HER2+) and triple negative (TNBC/ER−, PR−, HER2−) [3,4]. 

**Figure 1:**
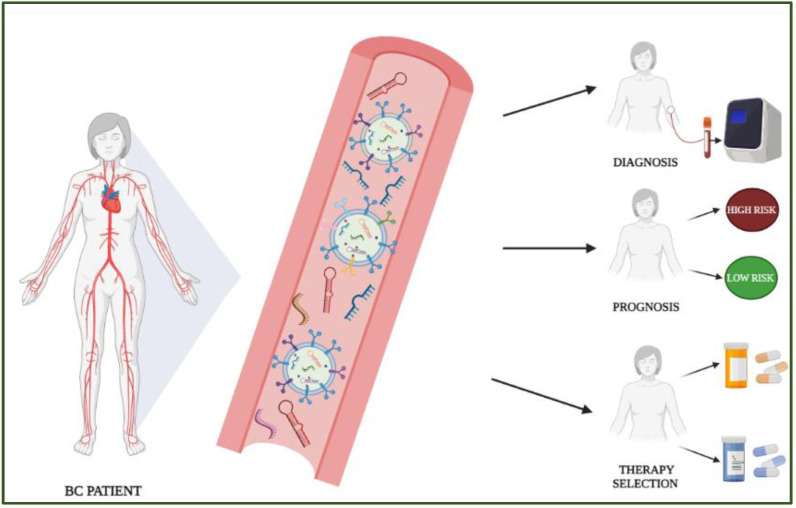
Schematic representation of the possible applications of miRNAs for BC patient management.

Despite the great advances in prevention and treatment, including the use of target and immuno-therapies, new tools are still required to identify women at higher risk of developing this disease, access the best therapeutic strategies and monitor the onset of therapy resistance. Among the different strategies for better management of BC patients, liquid biopsy has become a powerful and minimally invasive tool for repeatable biomarker testing [5]. In the plethora of circulating biomarkers retrievable from liquid biopsy, microRNAs (miRNAs) have emerged as very promising due to their higher expression in cancer patients compared to controls [6], their involvement in neoplastic formation and progression [7], and their stability to RNase, in a free form or entrapped in circulating extracellular vesicles (EVs) [8] (Figure 1). 

This review aims to summarize the insights of circulating miRNA analyses in the context of BC, both at a preclinical and clinical level, and to critically discuss the limitations and the possibilities of their implementation in a clinical context.

miRNAs are a class of small non-coding RNAs, with an average length of 22 nucleotides, identified in the early 2000s in vertebrates, flies, worms, plants and viruses [9]. Since then, several studies have been published reporting the participation of miRNAs in crucial biological processes [10]. Most miRNAs are transcribed from DNA sequences into primary miRNAs (pri-miRNAs), and processed into precursor miRNAs (pre-miRNAs) and, finally, mature miRNAs [11]; they can also interact with the 3′ UTR of target mRNAs to suppress gene expression [12]. However miRNAs can also interact with other regions, including the 5′ UTR, the coding sequence, and the gene promoters [13]. miRNAs bind transcripts with complementary sequences, leading to either mRNA degradation or translational suppression, even if their preferred mode of regulation among different cell types has not been fully elucidated [14]. Almost one decade ago, various literature reports demonstrated that miRNAs and miRNA–associated protein complexes could also stimulate gene expression post-transcriptionally via direct or indirect mechanisms [15]. The final outcomes of these additional properties can vary from fine-tuning effects to significant alterations in gene expression. In this context, recent evidence indicates that miRNA-mediated post-transcriptional up-regulation is selective, sequence-specific, and associated with the miRNA-containing ribonucleoprotein (miRNP) factors and other RNA-binding proteins [16].

It has been reported that the cell cycle state can impact miRNA-mediated gene regulation [17]. For example, the expression of GW182, a crucial protein for miRNA-mediated downregulation, was reported to be decreased in the G0 state, thus missing its interaction with the nuclear Argonaute (Ago) protein and allowing another protein, Fragile-X mental retardation protein 1 (FXR1), to be involved in the miRNP complex. These events result in miRNP-mediated gene activation [18]. Moreover miRNAs act coordinately with transcription factors involved in cell cycle regulation such as c-MYC, E2F or p53 [19,20,21,22]; this not only enhances the function of these factors, but also protects cells from replicative stress, limiting the excessive translation of the cell cycle proteins upon mitogenic or oncogenic stimuli.

Free circulating miRNAs have been found in all body fluids (such as saliva, serum, plasma, urine and milk) [23,24,25,26], thus suggesting possible non-cell-autonomous functions, and can be secreted as a result of either passive or active events [27]. Indeed, miRNAs are produced via passive leakage of cells exposed to injury, inflammation, apoptosis or necrosis, or by platelets that are characterized by a short lifespan [28,29,30]. Moreover, miRNAs can be actively secreted by a protein–miRNA complex [12], and may be found in the blood stream complexed with proteins [31,32] or carried within extracellular vesicles [33,34]. Cell-free circulating miRNAs are extremely stable and, thanks to their characteristics and molecular functions, considered potential clinical, non-invasive biomarkers for many pathologies. 

It has been found that their that their quantity can fluctuate among individuals, and their levels are often altered in patients [35]. This is in line with the growing idea that one of the most promising roles of miRNAs is to act as potential biomarkers. This possibility has been investigated in various medical fields. In this context, the diagnostic and prognostic utility of circulating cell-free miRNAs (cf-miRNAs) in human serum and plasma has been reported by several groups [36]. These investigations opened up the possibility of applying single cf-miRNAs or cf-miRNA signatures in screening programs to improve the early detection of cancer [35] or to be used to monitor disease progression. 

This review summarizes the pre-clinical and clinical implications of miRNAs in the field of BC.

### 1.1. Free Circulating miRNAs: Pre-Clinical Studies

Pre-clinical studies evaluating alterations in miRNA expression and function associated with different BC subtypes is based on the use of either in vitro human cancer-derived cell lines or mouse xenograft models. A systematic review focused on the advancements in pre-clinical imaging to study cell-free miRNAs in vitro and in mouse models of BC has recently been published [37]. 

In this review, few examples of the most recent studies dealing with miRNA modulation that takes advantage of either in vitro BC cell lines or in vivo models are reported. In this context, an interesting paper by Emma Gervin and colleagues indicates that the over-expression of miR-526b/miR-655 in poorly metastatic BC cell lines promotes an aggressive cancer phenotype both in vitro and in vivo, and their expression is significantly modulated by hypoxia [38].

Recently, it has also been proposed that the miRNA-long non-coding RNA (lncRNA) interaction is critical for miRNA-related gene expression. Two recent papers show that two lncRNAs, metallothionein 1J (MT1JP) and the hepatocyte nuclear factor 1 homeobox A-antisense RNA 1 (HNF1A-AS1), target specific miRNAs, playing a role in the tumorigenic process [39,40]. In the first, the authors demonstrated a significant negative correlation between MT1JP and miRNA-214, whose expression enhances the invasion ability of the BC cell lines MCF-7 and MDA-MB-231; in the second, HNF1A-AS1 physically targeted the pro-tumorigenic miR-20a-5p. 

An over-expression of miR-34a was found in BC tissues and BC cell lines in comparison to non-tumor tissues and cell lines [41,42]. miR-34a modulates the wnt/β-catenin pathway in non-metastatic MCF-7 cells, triggering a decrease in their proliferation, invasion and migration, both in vitro and in vivo [43].

Another interesting manuscript shows that miR-941 is significantly upregulated in metastatic MDA-MB-231 cells in comparison to the human breast epithelial cell line MCF-10A. Indeed, transfection of the miR-941 inhibitor significantly decreased the proliferation and migration of MDA-MB-231 cells by altering the expressions of p21, Cyclin D1, PP2B-B1, E-cadherin and MMP-13 [44].

The mouse xenograft models represent critical steps in translating results obtained in vitro. However, different aspects have to be carefully pondered when developing xenografts and comparing literature data, such as the use of a cell line or patient-derived tumor cells, the engraftment route, and the choice of the mouse strain. This latter aspect is particularly important to obtain efficient cell engraftment, since the immunodeficient strains generally used present immunological differences [45]. Recently, the NOD/Shi-scid/IL-2Rγnull (NOG) mice, derived from the NOD/SCID strain with a common gamma chain, has been introduced to efficiently obtain patient-derived xenografts [46]. Due to their immunologic characteristics—such as the lack of functional T and B lymphocytes, the multifunctional defects in NK activity, macrophage and dendritic cell function, and complement activity—NOG mice are considered the most appropriate host mouse models for direct xenografting of fresh tumor tissue [47].

miR-21 has been reported to be up-regulated in many cancers, including BC [48]. A recent paper reported the role of miR-21 in BC initiation, progression and treatment response, using two TNBC cell lines (4T1 and MDA-MB-468 cells) [49]. The role of miR-21 in breast cancer initiation was evaluated in mouse models by implanting wild-type miR-21 BC cells. Tumors were unable to grow in the mammary fat pads of mi-R21−/− mice, and grew in about 50% of heterozygous and 100% of miR-21 wild-type mice. The global ablation of miR-21 significantly decreased the tumorigenesis and metastasis of both mouse and human TNBC cell lines, suggesting that targeting miR-21 alone or in combination with various therapies could represent a valuable therapeutic approach for the future treatment of BC patients [49]. 

The evidence that miR-27b-3p could promote BC growth by inhibiting peroxisome proliferator-activated receptor gamma (PPARG) expression has been experimentally demonstrated in vivo by implanting MDA-MB-231 cells transfected with miR-27b-3p, miR-27b-3p mimics or miR-27b-3p mimics and treated with PPARG agonist [50].

Xenografts have been also used as models to test the anti-tumoral effects exerted by some miRNAs. In this context, the use of miR-497 for BC therapy has been verified in male athymic Balb/c nu/nu mice transplanted with MCF-7 cells: (i) wild-type, (ii) transfected with the negative control or iii) transfected with the miR-497 mimics; this demonstrated that it was able to inhibit tumor growth and angiogenesis, downregulating the expression levels of VEGF and HIF-1a [51]. Zhu and colleagues reported that miR-544 was down-regulated in the TNBC cell lines (HCC38, HCC1143 and HCC1395) and suppressed tumor growth in vivo, using a mouse xenograft tumor model performed using five-week-old athymic nude mice [52]. Similarly, the over-expression of miR-490-3p in a BC mouse xenograft model was demonstrated to suppress tumor growth induced by MCF-7 subcutaneously implanted in Balb/c nude mice [53].

Patient-derived xenograft (PDX) models are emerging as great tools to predict drug efficacy and understand the characteristics of many tumor types, including breast cancer; these models are also used to investigate the role of miRNAs as circulating biomarkers.

PDX models were efficiently used to detect miR-93 as a powerful suppressor of BC metastatic ability in the liver [54], or to identify miR-766 as a potent regulator of cancer metastatic behavior. Interestingly, miR-766 affected the metastasis process, thus representing a possible therapeutic target to counteract metastasis formation [55].

Although PDX models possess notable advantages compared to classical xenografts, their pre-clinical use in the field of breast cancer is still limited due to the difficulties in establishing and obtaining tumors that continue to progress and metastasize, thus resembling the patterns of the disease course observed in patients.

### 1.2. Extracellular Vesicle (EV)-Associated Circulating miRNAs: Pre-Clinical Studies

Recent evidence indicates that miRNAs can be present in the extracellular space, packaged into various membrane-bound vesicles, namely, extracellular vesicles (EVs), that could be released by the primary tumors. EVs can be roughly divided into three main types according to their size and biogenesis: exosomes, microvesicles and apoptotic bodies [56]. Exosomes are considered small EVs, originating from the endosomal compartment and possessing a diameter ranging from 30 to 130 nm [57]; microvesicles are a class of larger EVs, ranging from about 150 to 500 nm, and originating from the budding and fission at special lipid-raft domains of the plasma membrane [58]. Finally, apoptotic bodies are vesicles containing parts of a dying cell, bigger in size and formed during the execution phase of the apoptotic process [59]. Since consensus has not yet emerged on specific markers of EV subtypes [60], and miRNAs have been reported to be associated with the different EV species [61], we will generically refer to EV-associated miRNAs.

miRNAs within EVs are protected from serum RNases and are, therefore, particularly stable [62]. Several studies reported that miRNA levels, and in particular, EV-miRNA levels, in the blood correlate with the clinical classification and prognosis of certain tumors, making them potentially useful as cancer biomarkers [63,64]. In this context, in 2014, a pioneer study reported that the miR-200 family’s abundance in EVs is correlated with metastasis and relapse in BC [65]. Interestingly, the authors demonstrated that EV-miRNAs were not just circulating biomarkers, but were functional, promoting metastasis in vivo. Indeed, they showed that metastatic 4T1 cells, but not poorly metastatic 4TO7 cells, secreted miR-200 in EVs, and that 4TO7 cells were able to take up miR-200 from 4T1 EVs and become metastatic in a miR-200-dependent manner [65]. 

EV-miRNAs have been also investigated due to their interactions with other cell types comprising the tumor microenvironment. In this context, miR-125b has been extensively studied since it is one of the most abundant miRNAs secreted by mouse TNBC cell lines, and its delivery within EVs is responsible for fibroblast activation in mouse tumor models [66]. Similarly, miR-370-3p, contained in BC-derived EVs, was described to induce fibroblast activation by downregulating cylindromatosis (CYLD) expression and activating the NF-κB signaling pathway [67].

In 2020, an interesting paper reported that the Mitochondrial Calcium Uniporter (MCU), which has been suggested to be involved in BC progression, was negatively correlated with miR-4488 in EVs derived from BC cells. Indeed, miR-4488 has been described to suppress angiogenesis via direct targeting of CX3CL1 [68].

Preclinical studies indicate that miR-182-5p is enriched in EVs from metastatic MDA-MD-231 cells and enhances the proliferation, migration and angiogenesis of HUVECs both in vitro and in vivo [69]. 

Since tumor-derived EVs carry a pro-tumorigenic cargo, often in the form of miRNAs, it has been proposed that the inhibition of EV secretion or uptake, as well as the blocking of specific EV components, could represent a novel strategy for cancer therapy [70,71]. 

### 1.3. Free Circulating miRNAs: Clinical Studies

Considerable attention and an enormous amount of effort and resources have been dedicated to biomarker discovery and validation in liquid biopsies from BC patients. One of the first observational studies dealing with circulating miRNAs as minimally invasive biomarkers for BC was published in 2010 [72]. The authors collected 127 blood samples, including 83 BC patients and 44 healthy age-matched female volunteers. The extraction of miRNAs, followed by quantitative real-time PCR, revealed that the expression of mir-195 and let-7a were significantly increased in the blood of HER2 positive compared to HER2 negative BC patients and to healthy controls. In this work, mir-21 and mir-10b were reported to be significantly increased in patients with ER-negative disease [72]. Starting from this paper, a huge number of manuscripts have also been published on this topic, thanks to the advancement in technologies related to miRNA isolation and analysis. Nowadays, many studies involve the enrollment of BC patients at initial diagnosis, during neoadjuvant chemotherapy, and after the tumor resection. In this context, it has been recently reported that the expression of mir-21, mir-181a and mir-10b is significantly increased at initial locally advanced BC diagnosis, whereas that of mir-145 and let-7a is significantly decreased, compared to healthy individuals [73]. Interestingly, the diagnostic accuracy of mir-21 was greater than other diagnostic markers, and, after the end of the treatment, its expression returned to control values. 

mir-10b and mir-21 have been confirmed to be highly expressed in BC [35,48,74]. In particular, mir-10b directly targets the HOXD10 and Krüppel-like factor-4 genes [75] and is upregulated in metastatic primary tumors [76]. mir-21 has several targets including tropomyosin 1α and programmed cell death 4 (PDCD 4), and its upregulation is associated with tumor progression and a poor prognosis [77,78]. A recent meta-analysis [79], taking into account seven articles for a total of 1629 BC cases, pointed out the predictive value of miR-21 expression level in both BC tissue and plasma samples, suggesting that it could be used as prognostic biomarker in BC. 

Another miRNA frequently deregulated in BC is miR-155, which is overexpressed in the serum of BC patients compared with healthy individuals. It is involved in different signaling pathways related to disease progression [80], since it negatively regulates, among others, cytokine signaling 1 and fork-head box O3a [81]; moreover, it targets BRCA1 [82], which is involved in cell cycle progression and in DNA repair. miR-155 has been shown to be a reliable biomarker for TNBC, since its expression is associated with ER/PgR/HER2 expression [83]. In addition, it was found to be overexpressed with miR-10b and miR-195 [84]. Notably, unlike other circulating miRNAs, the overexpression of miR-155 is often concordant across different studies, making it a specific biomarker candidate for BC. On the contrary, different results are reported for other miRNAs. For example, miR-10 [6] and miR-34 have been found to be over-expressed in BC [83], while other authors reported no association with BC [72] or downregulation [84], respectively.

Another group of miRNAs that is often deregulated in BC is the mir-200 family. This family, composed of five miRNAs, has a tumor-suppressor role and it is organized in two clusters: cluster I (mir-200b/22a/429) and cluster II (mir-200c/141) on chromosomes 1 and 12, respectively [85]. The expression of these clusters is suppressed during epithelial-mesenchymal transition, a mechanism that supports BC motility and invasiveness [86]; accordingly, miR-200c modulation in breast cancer cells affects cell migration and invasion [87]. 

Several studies focused not only on upregulated miRNAs, but also on down-regulated ones in BC. Among the others, miR-335 inhibits metastasis by targeting the transcription factor Sry-box 4 and the protein tenascin-C, acting as a tumor suppressor. This action involves a reduction in cell viability and the promotion of apoptosis [88]. Indeed, a loss of miR-335 leads to the acquisition of metastatic properties [89].

Recently, a paper has been published reporting that mir-301a, known to function as an oncogene in many human cancers, should be considered as a novel biomarker associated with decreased overall survival [90]. The authors demonstrated, using an in situ hybridization (ISH)-based classification system on 380 samples of BC tissue, collected at the time of diagnosis, that miR-301a expression could identify patients at higher risk of relapse.

Cuk K. and colleagues reported the diagnostic potential of a panel composed of seven circulating miRNAs (miR-127-3p, miR-148b, miR-376a, miR-376c, miR-409-3p, miR-652 and miR-801) in two cohorts of stage I and II BC patients, suggesting that they can be used for early diagnosis [91].

In another study, 43 circulating miRNAs were differentially expressed between BC patients and healthy controls, with patients exhibiting higher miR-148b, miR-133a and miR-409-3p levels [92]. A technology based on Taqman low density array (TLDA) cards was used to analyze serum miRNAs in 39 early BC patients compared to 10 healthy volunteers [93]. Among the 17 miRNAs identified, 14 were evaluated in the validation cohort. mir-484 was found to be significantly over-expressed in BC serum compared to healthy volunteers, suggesting that it could be useful as an adjunct to mammography for early BC detection.

Circulating miRNAs have been demonstrated to have prognostic and predictive value in several studies. The majority of these were focused on TNBC, the most aggressive subtype of BC, and proposed different signatures of circulating miRNAs associated with poor clinical outcomes. Zeng and colleagues showed, in a cohort of 173 TNBC women, that miR34a-b-c was significantly less expressed in these patients compared to healthy women, and these miRNAs were associated with tumor progression and indicated worse prognosis [94]. Other authors performed genome-wide miRNA expression and real-time PCR analyses in the serum of 60 primary TNBC patients. Then, they validated, in an independent cohort of 70 TNBC cases, a miRNA signature (miR-18b, miR-103, miR-107, and miR-652) that predicted tumor relapse and poor overall survival [95]. Another signature of the seven miRNAs (miR-21-5p, miR-375, miR-205-5p, and miR-194-5p, upregulated, and miR-382-5p, miR-376c-3p, and miR-411-5p, downregulated) indicated recurrence in TNBC, but also in hormone-receptor-positive (HR+) BC patients [96]. Circulating miRNAs are also promising in advanced settings, where they have been found to be related to tumor progression and metastases [83]. 

Several miRNAs or miRNA signatures have been identified to be correlated with a response or resistance to the therapy. Recently Di Cosimo et al. [97], analyzing the data from the NeoALTTO trial, conducted in early BC setting, were able to demonstrate the signature of four miRNAs that discriminate between patients with different responses to HER2-targeted therapy (trastuzumab lapatinib and/or trastuzumab-based therapy). 

In another study, miR-19a and miR-205 were found to be predictive of chemosensitivity for luminal A (LA) BC patients treated with neoadjuvant Epirubicin plus Paclitaxel therapy [98]. 

The emergence of drug resistance could also be monitored by circulating miRNAs. Wang H. and co-workers [99] reported high levels of miR-125b in the serum of non-responder BC patients treated with an association of 5-Florouracil (5-FU), Epirubucin and Cyclophosphamide (FEC). Other authors showed, in HER2+ metastatic BC patients, that a serum-based miRNA signature (miR451a, miR-16-5p, miR-17-3p and miR-940) can effectively distinguish patients who are sensitive to first-line trastuzumab plus chemotherapy from the resistant ones [100]. In this paper, Li H. and co-workers were also able to determine the specific cellular origin of the four predictive serum-derived miRNAs, thus correlating their expression with their functional role and involvement in the mechanism underlying trastuzumab resistance; intriguingly, their data suggest that sensitivity to trastuzumab could be mediated by the immune system via EV-associated miRNA regulation.

As reported above and in Table 1, many other studies have been published on circulating miRNAs and their possible application as BC biomarkers. However, one of the main limitations of the above-mentioned studies is that in most of them, the origin of the identified miRNAs has not been verified, and the correlation of their expression with the corresponding BC tissue has not been analyzed. For this reason, global miRNA expression profiling using paraffin-embedded tissues from BC patients and samples from healthy controls have been compared to expression levels in serum/plasma, revealing that the expression of several miRNAs, such as miR-505-5p, miR-125b-5p, miR-21-5p miR-96-5p [101], or mir-182 [102], is significantly up-regulated in BC patients compared to healthy controls, and matches with the corresponding tumor tissue. On the contrary, some studies reported differences between the signature obtained by analyzing miRNA in plasma/serum and in tissue. Minghui Li et al. [103] identified five plasma miRNAs (let-7b-5p, miR-122-5p, miR-146b-5p, miR-210-3p and miR-215-5p) whose expression levels were significantly different in a cohort of 257 BC patients and 257 healthy controls. This miRNA signature was further evaluated among 32 pairs of BC tissues and the adjacent normal tissue samples, as well as plasma-EV samples, obtaining different results. In tissue biopsies, let-7b-5p was contrarily down-regulated, and in EV samples, only miR-122-5p was significantly up-regulated, as in BC plasma. These data underline that the miRNA source should be carefully evaluated. 

### 1.4. EV-Associated Circulating miRNAs: Clinical Studies

In Table 2, some of the EV-miRNAs identified as potential biomarkers in a clinical setting are reported. The pioneer study published in 2016 compared miRNAs enriched in EVs from BC cell lines with the levels of the more abundant miRNAs in plasma-derived EVs [112]. Interestingly, these authors found a corresponding overexpression of miR-21 and miR-1246 in plasma, suggesting, for the first time, a panel of EV-miRNA for BC diagnosis.

A more recent study by Li M and colleagues compared circulating free-miRNAs and EV-miRNAs isolated from the plasma, serum and tumor tissue of BC patients and heathy controls [108]. The authors identified two different signatures consisting in four free miRNAs in plasma (miR-20b-5p, miR-92a-2-5p, miR-106a-3p and miR-106a-5p) and four miRNAs in serum (miR-19b-3p, miR-20b-5p, miR-92a-3p, and miR-106a-5p) that were overexpressed in BC, thus showing a great potential to discriminate BC patients. These miRNAs were further evaluated in matched the tissue samples and extracellular vesicles extracted from plasma and serum. This comparison put in evidence that only two miRNAs (miR-20b-5p and miR-106a-5p) exhibited the same pattern in tissue as the circulating free miRNAs, while the expression of EV-miRNAs from plasma and serum, with the exception of EV-miR-20b-5p, was concordant with the ones from circulating free-miRNAs. These findings, similarly to the results reported above [103], underline the different miRNA patterns expression in tissue compared to that detected in the blood stream, thus suggesting a careful evaluation of the biological sources. The partial concordance between the behavior of miRNAs extracted from tissue with respect to the circulating ones is peculiar; this is because the correlation of specific biological features between tumor tissue and other potential biomarkers that are retrievable by liquid biopsy are the basis of their analysis as a minimally invasive surrogate of the primary tumor. 

EV-associated miRNAs have also recently been proposed as promising non-invasive diagnostic biomarkers for inflammatory breast cancer (IBC) [113]. In particular, miR-181b-5p and miR-222-3p were significantly upregulated, whereas let-7a-5p was downregulated in IBC patients. However, bioinformatic analysis indicated that the diagnostic accuracy of let-7a-5p alone was the highest for IBC.

The use of EV-miRNAs as prognostic biomarkers for tumor recurrence has been proposed. A study published in 2017 [114] showed 11 differentially expressed EV-miRNAs between recurrent and non-recurrent patients, and four miRNAs (miR-17-5p, miR-93-5p, miR-130a-3p, and miR-340-5p) were significantly associated with recurrence. 

The possibility of using EV-associated miRNAs as predictors of pathological complete response (pCR) has been also investigated in the plasma samples of 20 BC patients treated with neoadjuvant chemotherapy [115]. The miRNA profiling determined by RNA sequencing indicated that three miRNAs (miR-30b, miR-328 and miR-423) were able to predict pCR before neoadjuvant chemotherapy. The analysis after the first chemotherapy dose indicated that only miR-141 was able of predicting pCR, while miR-34a and miR-182 encapsulated in EVs predicted non-pCR.

The possibility of analyzing EV-associated miRNAs to discriminate patients with pCR and non-pCR has been also investigated in a recent paper published in Oncology Letters [116]. A combined signature of four miRNAs (miR-4448, miR-2392, miR-2467-3p and miR-4800-3p) was identified to be used to discriminate between pCR and non-pCR patients with TNBC.

Surprisingly, in a study that considered 27 BC patients and three healthy controls, no significant differences were observed among basal-like, HER-2+, luminal A, luminal B and healthy control groups [117]. A significant EV-miRNA deregulation was, instead, observed in TNBC patients compared to healthy volunteers, and three of them—miR-150-5p, miR-576-3p and miR-4665-5p—were able to distinguish breast cancer patients with recurrence from those without recurrence.

miR-16 and miR-30b packaged inside EVs were able to distinguish BC from ductal carcinoma in situ (DCIS) [118], with miR-16 particularly enriched in estrogen-positive patients and miR-30b expression inversely proportional with recurrence. EV-miR-93 was, instead, up-regulated in DCIS patients.

In another manuscript published in 2019, the authors reported that before neo-adjuvant therapy, the expression of EV-miR-21 and EV-miR-105 was significantly higher in metastatic BC patients compared to non-metastatic or healthy controls [119]. In the same paper, the authors also reported that another EV-miRNA, namely miR-222, was able to discriminate the basal-like and luminal B from luminal A tumor subtypes [119].

In 2017, an elegant paper was published focusing on FoxP3-inducible breast cancer cells and Foxp3 heterozygous Scurfy mutant (Foxp3 sf/+) female mice to identify a FOXP3-KAT2B-miR-200c/141 axis [120]. A higher plasma level of EV-associated miR-200c and miR-141 was found in patients with metastatic disease compared to patients with benign or locally advanced BC, or to healthy controls, suggesting that these two miRNAs are potential biomarkers for the early detection of breast cancer metastases.

EV-miRNAs could also be specific of BC subtypes. As an example, patients with Luminal A (LA) disease showed a higher expression of the EV-miR-142-5p and miR-320a compared to controls, while the expression level of miR-150-5p discriminated LA and TNBC subtypes with accuracy [121]. The same study also reported that higher levels of miR-142-5p and miR-320a were associated with tumors smaller than 20 mm.

Checking blood biomarkers could also be correlated with therapy response, drug resistance, and survival. An altered EV-miRNA signature has been associated with neoadjuvant chemotherapy [122], and tamoxifen-resistance in ER+ tumors [123].

Another interesting study reports that 12 miRNAs (miR-138-5p, miR-210-3p, miR-423-5p, miR-574-3p, miR-744-5p, miR-3178, miR-4258, miR-4298, miR-4443, miR-6780b-3p, miR-7107-5p, miR-7847-3p) are up-regulated after neoadjuvant chemotherapy, potentially indicating drug resistance in patients with BC [124]. Considering these few examples, chemotherapy seems to influence miRNA levels, particularly EV-miRNA, and it is also frequently associated with drug resistance.

## 2. Discussion

This review recapitulates just a few of the many reports dealing with circulating free or EV-associated miRNAs in the context of breast cancer. Starting from an overview of preclinical studies performed in vitro, with cancer cell lines or in vivo, using BC xenograft models, we then summarize some of the results obtained from analyzing the liquid biopsies of BC patients. Depending on the selection of the cohort of patients and related healthy controls, miRNAs have been studied as potential useful diagnostic, prognostic biomarkers.

Despite the huge amount of work and the resources dedicated to this topic, including the conductions of clinical trials (some of them cited in Table 3), several limitations still hamper the identification of reliable promising miRNAs to be used as biomarkers or drug agents, and their consequent translation to a clinical setting. Some discrepancies among different studies can be caused by the heterogeneity of breast cancer. From this perspective, a more precise patient inclusion criteria or enlarging of the cohort size could overcome the differences in miRNA expression caused by the intrinsic heterogeneity of the disease. Patients’ treatment should be also taken into account, since miRNA levels could be heavily affected by various pathological conditions or comorbidities (such as inflammation or cardiovascular diseases), but also by physiological conditions, such as diet or physical activity. As a consequence, the selection of internal controls should also be carefully evaluated within a single investigation. The most recent studies are, indeed, more focused on a precise BC subtypes—in particular, TNBC or HR+ BC—or are conducted in specific therapeutic settings; this, in part, minimizes the possible interferences mentioned above.

Moreover, every step, from sample collection to processing and analysis, should be precisely reported in order to be able to make reasonable comparisons among studies. Most of the studies use serum or plasma indiscriminately. A recent paper has demonstrated that serum can be considered as a better choice, and circulating miRNA levels are higher in serum than in plasma; however, it has been suggested that miRNA release in serum could partly be a consequence of the coagulation processes, independently to the disease [108]. In addition, potential interference by platelets and white blood cells during sample preparation should be considered [129]. Moreover, in the cited study by Li and colleagues, only plasma-associated, but not serum-associated, miRNAs were found to be correlated with clinical features [108]. Therefore, it is important to consider the same material, avoiding samples with clear signs of hemolysis, and use standardized protocols. In addition, preclinical conditions, including processing time, should be standardized in order to avoid alterations in miRNA profiles. The best, as optimum practice in liquid biopsy, would be rapid processing of the plasma after blood withdraws, or serum separation within 1 h of the sample collection. Alternatively, commercial, dedicated preservative-containing tubes could be used, but the effect of these preservatives on miRNA recovery has not been extensively evaluated. 

Another important limitation lies in the low miRNA levels in the blood stream. This makes them particularly difficult to identify using standard techniques. Several strategies have been proposed, such as an enrichment step before global expression profiling [130]. Different considerations should be done with regard to the platform applied to miRNA evaluation. The most-used is reverse-transcription quantitative PCR (RT-qPCR), which has the advantage of being very high-throughput and sensitive, but is limited to the analysis of known miRNAs. Moreover, the required amplification steps, which are currently performed using different strategies, could affect miRNA detection. A more promising approach is Next Generation Sequencing (NGS), which allows the detection of a larger panel including novel miRNAs with high sensitivity; however, it is more expensive, and requires time-consuming protocols and a high amount of starting material. Therefore, most of the recent studies have been conducted by combining workflows, using microarray or NGS for a wider miRNA detection followed by a validation phase based on qRT-PCR, or alternatively, digital PCR. The use of complementary technical approaches would make miRNA identification more robust. 

Although EVs could represent an interesting and complete source of circulating miRNAs, no definitive consensus has been reached so far on EV isolation methods [60]. This aspect represents the main limitation in the translation of EV-related studies into the clinic.

The data and the selected studies reported in this review, even if limited, support the potentialities of the implementation of circulating miRNAs for the management of BC patients. However, even if some miRNAs (such as the mentioned miR-21 or miR-155) or miRNA signatures showed a diagnostic or predictive/prognostic value, there are still some disagreements; this suggests that their role deserves further evaluation. For example, miR-21 and miR-155, the most promising miRNAs in BC, have also been demonstrated to not be BC-specific, as they are associated with other tumors or diseases [131]. These authors conducted an extensive and critical analysis of the literature, identifying 281 mRNAs associated with BC, 216 deregulated in tissue, and 163 in the blood stream. They put in evidence that the most-studied miRNAs (miR-155, miR-21, miR-195 and miR-145), frequently suggested as potential biomarkers for BC, are, with the exception of miR-195, the most non-specific. The same authors confirmed such findings in a more recent systematic review focused on the prognostic significance of miR-155 [132]. Such data show that the identification of reliable and clinically useful circulating miRNAs needs to be verified. The correlation of miRNA expression with other “circulome biomarkers”, like circulating tumor cells [119] or circulating tumor DNA, could also provide a multiparametric approach to, together with the standardization of the pre-analytical and analytical methodologies, overcome the challenges that still prevent miRNA applicability in clinical practice. 

## 3. Conclusions

Breast cancer is a highly heterogeneous disease, and novel, minimally invasive tools are still needed to better characterize the different subtypes. With this perspective, circulating miRNAs are promising breast cancer biomarker candidates. In this review, a brief description of the state-of-the-art of miRNA research in breast cancer is reported. Single miRNAs or miRNA signatures have been validated as diagnostic and prognostic/predictive in BC, both in early and advanced settings. However, very little consensus emerged from the different studies; this is primarily due to the selection of patients included in the different studies, but mostly due to the different experimental approaches used. All these issues have, so far, prevented the implementation of miRNA analysis in a clinical context. Nevertheless, more accurate trial designs, including better patient-selection and the standardization of analytical approaches, could overcome such limitations.

## Figures and Tables

**Table 1:** Circulating miRNA as potential diagnostic, predictive and prognostic biomarkers.

miRNA (Expression)	Cohort	Sample	Clinical Significance	Ref
miR296-3p, miR-575, miR-3160-5p, miR-4483, miR-4710, miR-4755-3p, miR-5698, miR-8089 (new MBC) miR-8089 and miR-5698 (↓ OS)	MBC (*n* = 147)	Serum	Predictive, Prognostic (new distant metastasis, OS)	[104]
miR-331 (↑), mir-195 (↓ LA-MBC) miR-331 (↑), mir-181a (↓ BC) miR-181a (↑ HCs)	LA (*n* = 31), LA-MBC (*n* = 22), HCs (*n* = 21)	Whole blood	Diagnostic (MBC vs. luminal A)	[105]
miR-21, miR-181a, miR-10b (↑) miR-145 and let-7a (↓)	BC (*n* = 30), HCs (*n* = 20)	Plasma	Diagnostic, Predictive: (initial LABC, PFS)	[73]
miR-21, miR-23b, miR-200b, miR-200c (↑ MBC) miR-21, miR-190, miR-200b, miR-200c (↑ MBC)	Early BC (*n* = 133), MBC (*n* = 110)	Plasma	Diagnostic, Prognostic (MBC vs. Early BC; miR-200b ↑ → ↓ OS; miR23b and miR-190 ↑ → ↓PFS)	[106]
miR-1246, miR-206, miR-24, miR-373 (↑ BC)	BC (*n* = 226), HCs (*n* = 146)	Plasma (Serum)	Diagnostic (early-stage BC detection)	[107]
miR-106a-3p, miR-106a-5p *, miR-20b-5p *, miR-92a-2-5p miR-106a-5p *, miR-20b-5p *, miR-19b-3p, miR-92a-3p miR-106a-5p *, miR-20b-5p * (↑)	BC (*n* = 200), HCs (*n* = 200) ** BC (*n* = 204), HCs (*n* = 202) ** Plasma: BC (*n* = 32), HCs (*n* = 32) ***; serum: BC (*n* = 32), HCs (*n* = 32), ***; tissues: BC (*n* = 32), HCs (*n* = 32) ***	Plasma Serum Plasma, Serum, Tissues	Diagnostic, prognostic (BC vs. HCs; association with clinical/pathological featuresDiagnostic (BC vs. HCs)	[108]
miR-210 (↑ BC) miR-152 (↑ BC)	BC (*n* = 30), BBC (*n* = 5), HCs (*n* = 5)	Plasma	Diagnostic, prognostic: early diagnosis, staging	[109]
miR-24-3p (↑ MBC)	BC (*n* = 115); MBC (*n* = 115)	Plasma	Prognostic, predictive (metastasis, OS)	[110]
miR-19a/b-3p; miR-25–3p; miR-22-3p; miR-93-5p; miR-210-3p (↑), miR-199a-3p (↓)	TNBC (*n* = 36), LA (*n* = 16), LB (*n* = 41), HCs (*n* = 34)	Plasma	Predictive, prognostic (drug-resistance in TNBC; OS)	[111]
Lapatinib: miR-376c-3p, miR-874-3p; miR-197-3p, miR-320c, miR-100-5p (pre); miR-144-3p, miR-362-3p, miR-100-5p (post) Trastuzumab: miR-374a-5p, miR-574-3p, miR-140-5p, miR-328-3p, miR-145-5p (post) Lapatinib+Trastuzumab: miR-34a-5p, miR-98-5p, miR-100-5p (post)	HER2^+^ BC (*n* = 429)	Plasma (pre/2 wks post NAC)	Predictive, prognostic (pCR-NAC response)	[97]

Abbreviations: BBC—benign breast condition; HCs—healthy controls; LA—luminal A; LABC—locally advanced breast cancer; LB—luminal B; MBC— metastatic breast cancer; NAC—neoadjuvant chemotherapy; OS—overall survival; pCR—pathological complete response; PFS—progression-free survival; ↓—downregulated; ↑—upregulated; * overlapping miRNAs in plasma, serum; ** 114 plasma–serum matched samples; *** plasma–serum–tissue matched samples.

**Table 2:** EV-miRNAs as potential diagnostic, predictive and prognostic biomarkers.

miRNA (Expression)	Cohort	Sample	Clinical Significance	Ref
miR-21-3p, miR 105-5p (↑ MBC pre-NAC); miR-222-3p (↑ basal-like, LB pre-NAC);	47 LBC (*n* = 47), MBC (*n* = 6), HCs (*n* = 8)	Serum	Diagnostic, predictive, prognostic (trastuzumab resistance)	[119]
miR-16 (↑BC, DCIS) miR-93 ((↑DCIS) miR-30b (↓ recurrence)	BC (*n* = 111), DCIS (*n* = 42), HCs (*n* = 39)	Plasma	Diagnostic, predictive (associated with clinical/pathological features, recurrence)	[118]
(a^#^) miR-142-5p, miR-320a, miR-4433b-5p (↑BC vs. HCs, LA vs. HCs); (b^#^) miR-142-5p and miR-320a (↑BC vs. HCs, LA vs. HCs); (c^#^) miR142-5p, miR-150-5p (↑LA vs. TNBC)	LA (*n* = 26), TNBC (*n* = 23), HCs (*n* = 26)	Serum	Diagnostic, prognostic: (BC vs. HCs, LA vs. TNBC; associated with clinical/pathological features)	[121]
miR-181b-5p, miR-222-3p (↑ IBC) let-7a-5p (↓ IBC)	IBC (*n* = 23), non-IBC (*n* = 34), HCs (*n* = 20)	Plasma	Diagnostic	[113]
miR-16, miR-27a, miR-27b, miR-143, miR-365 (↓ TNBC vs. Her2^+^) let-7g, miR-148a, miR-202, miR-335, miR-370, miR-376c, miR-382, miR-422a, miR-433, miR-489, miR-628, miR-652, miR-891a (↑ TNBC vs. Her2^+^) miR155, miR301 (best predictor pCR)	Her2^+^ BC (*n* = 211), TNBC (*n* = 224), HCs (*n* = 20)	Plasma	Diagnostic, predictive (associated with clinical/pathological features, pCR)	[125]
(a) miR-4448, miR-2392, miR-2467-3p and miR-4800-3p (↑ pCR+) (b) recurrence: 15 miRs ↑ (miR-195-5p: 4.43 fc, *p* = 0.02); 28 miRs ↓ (miR-548ab: 0.23 fc, *p* < 0.001)	TNBC: (a) pCR (*n* = 12), w/o pCR (*n* = 12) (b) w/o pCR: recurrence (*n* = 8), w/o recurrence (*n* = 8)	Serum pre ^a^/post ^b^ NAC	Predictive, prognostic (pCR+/pCR−; higher expression → longer OS)	[116]
Recurrence: miR-338-3p, miR-340-5p, miR-124-3p (↑ serum); miR-340, 195-5p, miR-17-5p, miR-93-5p, miR-130a-3p (↑ tumor) miR-340, 195-5p, miR-17-5p, miR-93-5p, miR-130a-3p (↑ tumor)	BC: recurrence (*n* = 16), w/o recurrence (*n* = 16) Tissues: recurrence (*n* = 35), w/o recurrence (*n* = 39)	Serum, tumor tissue	Predictive, prognostic (associated with recurrence)	[114]
miR-223-3p (↑IDC)	BC (*n* = 185), HCs (*n* = 146)	Plasma	Diagnostic (IDC vs. DCIS; associated with clinical/pathological features)	[126]
miR-1246 and miR-155 (↑Trastuzumab-resistant)	Early BC (*n* = 107), MBC (*n* = 68)	Plasma	Predictive, prognostic (trastuzumab: resistant vs. sensitive pt; EFS, PFS)	[127]
(a) miR-30b (↑), miR-328, miR-423 (↓pCR+) (b) exo-miR-141 (↓pCR+); miR-34a, miR183 (↑), miR-182 (↓pCR−)	IDC (*n* = 20)	Plasma (pre/2 wks post NAC)	Predictive (pCR to NAC)	[115]
miR-185, miR-4283, miR-5008 and miR-3613 (↓) miR-1302, miR-4715 and miR-3144 (↑)	TNBC (*n* = 34)	Plasma	Predictive (poor NAC responders)	[128]

Abbreviations: BC—breast cancer; DCIS—ductal carcinoma in situ; EFS—event-free Survival; fc—fold change; HCs—healthy controls; IBC—inflammatory breast cancer; IDC—invasive ductal carcinoma; LA—luminal A; LB—luminal B; LBC—localized breast cancer; MBC—metastatic breast cancer; NAC—neoadjuvant chemotherapy; OS—overall survival; pCR—pathological complete response; PFS—progression-free survival; TNBC—triple negative breast cancer; ↓—downregulated; ↑—upregulated; #—single and/or combined; pt—patients.

**Table 3:** The table illustrates clinical trials registered by 31 March 2022 (clinicaltrials.gov, searching for “miRNA” and “circulating microRNA” and “breast cancer” or “exosome” and “miRNA” and “breast cancer”).

NCT Number	Title	Study Type and Design	Brief Description	Enrolled Patients	Start–End Status	Country
NCT01612871	Circulating miRNAs as Biomarker of Hormone Sensitivity in Breast Cancer (MIRHO)	Interventional, Phase 4; Single group assignment; Prospective	Test in the advanced setting of the role of circulating MiRNA in comparison with tissue	39	June 2012 Jan 2016 Completed	France
NCT05151224	Circulating microRNA 21 Expression Level Before and After Neoadjuvant Systemic Therapy in Breast Carcinoma	Observational cohort; Prospective Diagnostic Test: microRNA21	miR-21 expression in early setting (before and after NAC)	40	Dec 2021 Jan 2024 Not yet recruiting	Egypt
NCT02065908	Circulating miRNAs as Biomarkers of Cardiotoxicity in Breast Cancer	Observational Cohort; Prospective	Circulating microRNA in serum as sensitive and specific biomarker of cardiotoxicity in cancer patients treated with anthracycline-based chemotherapy.	128	Jan 2014 Dec 2016 Completed	Poland
NCT01722851	Circulating miRNAs	Observational: Cohort Prospective	Circulating miRNA markers as predictive of neoadjuvant and adjuvant chemotherapy	255	May 2011 Dec 2021 Completed	Ireland
NCT04720508	Aberrant Expression of Micro RNA for Diagnosis of Breast Cancer	Observational: Case-control; Retrospective Diagnostic test: blood sample collection	Assess serum miRNA-373 and miRNA 425-5p, correlate with clinicopathological features	50	Dec 2021 Dec 2023 Not yet recruiting	Egypt
NCT03779022	miRNA and Relevant Biomarkers of BC Patients Undergoing Neoadjuvant Treatment	Observational cohort; Prospective Genetic: microRNA	Investigate miRNAs as predictive biomarkers of neoadjuvant therapy	100	Nov 2015 Dec 2019 Unknown	China
NCT03528473	Adapted Physical Activity (APA) in a Breast Cancer Population	Interventional; Randomized	Investigate miRNA levels in serum of post-menopausal, inactive patients in follow-up, w/wo HT hormone therapy after completed AR/AC therapy	100	Jan 2019 Dec 2022 Active, not recruiting	Italy
NCT04771871	MicroRNA Profiles in Triple Negative Breast Cancer (TARMAC)	Interventional, Phase 2; Single group	Assess blood miRNA and ctDNA levels during and after standard chemotherapy in black TNBC patients	42	Nov 2021 Aug 2023 Recruiting	Nigeria
NCT02618538	The Andromeda Study Predictive Value of Combined Criteria to Tailor BC Screening	Observational: Cross-Sectional	Investigate variance of plasma miRNAs associated with BC risk in HCs and BC patients	26,600	Jul 2015 Mar 2018 Completed	Italy
NCT03118882	STI.VI Study: How to improve Lifestyle in Screening Context (STIVI)	Interventional: Randomized; Prevention Biological sampling (blood and saliva)	Investigate deregulation of plasma miRNAs associated with breast/colorectal risk in HCs and cancer patients: screening as potential biomarkers (follow up to 10 years after study end)	1270	May 2020 June 2014 Completed	Italy
NCT03255486	Identification and Evaluation of Biomarkers of Resistance to Neoadjuvant Chemotherapy (IDEASEIN)	Interventional: Single group Assignment; Prevention; Blood Sample	Biomarkers of resistance to neoadjuvant chemotherapy in locally advanced breast cancer	164	Jul 2013 Dec 2016 Completed	France
NCT02605512	BreAst Cancer and Cardiotoxicity Induced by RAdioTherapy: the BACCARAT Study (BACCARAT)	Interventional: Single Group Assignment; Screening	Evaluate circulating biomarkers as predictive of whether 3DCRT induces cardiac toxicity	120	Oct 2015 Sept 2020 Unknown	France
NCT04781062	Development of a Horizontal Data Integration Classifier for Noninvasive Early Diagnosis of Breast Cancer (RENOVATE)	Interventional: non Randomized; Sequential Assignment; Blood and urine (T0-T1)	Develop a non-invasive molecular profile for early diagnosis, correlating blood (ctDNA, proteins, exosomes) and urine (ctDNA) biomarkers	750	Jan 2021 Dec 2024 Recruiting	Italy
NCT04530890	Interest of Circulating Tumor DNA in Digestive and Gynecologic/Breast Cancer	Interventional: Single Group Assignment; Basic Science; Blood Sample	Assess diagnostic, prognostic and predictive impact of ctDNA and exosomes in digestive and gynecological/breast cancers (pre and during treatment, progression and/or relapse, monitoring or treatment break)	1000	Jan 2021 Sept 2030 Recruiting	France

Abbreviations: ARAC—adjuvant radio/chemo; BC—breast cancer; TNBC—triple negative breast cancer; HT—hormone therapy; 3DCRT—three-dimensional conformal radiation therapy.
